# Diagnosis of Nonperitonealized Colorectal Cancer with Computerized Tomography Image Features under Deep Learning

**DOI:** 10.1155/2022/1886406

**Published:** 2022-05-25

**Authors:** Xiaohong Wang, Changyi Guo, Yufeng Zha, Kai Xu, Xiaochao Liu

**Affiliations:** ^1^Department of Critical Care Medicine, The Second Affiliated Hospital of Shaanxi University of Chinese Medicine, Xianyang 712000, Shaanxi, China; ^2^Image Center, The Second Affiliated Hospital of Shaanxi University of Chinese Medicine, Xianyang 712000, Shaanxi, China; ^3^Department of General Surgery, The Second Affiliated Hospital of Shaanxi University of Chinese Medicine, Xianyang 712000, Shaanxi, China; ^4^Department of Medical Imaging, Baoji Central Hospital, Baoji 721000, Shaanxi, China; ^5^Department of Gastroenterology, Shaanxi Hanzhong Centre Hospital, Hanzhong 723000, Shaanxi, China

## Abstract

This study aimed to explore the value of abdominal computerized tomography (CT) three-dimensional reconstruction using the dense residual single-axis super-resolution algorithm in the diagnosis of nonperitonealized colorectal cancer (CC). 103 patients with nonperitonealized CC (the lesion was located in the ascending colon or descending colon) were taken as the research subjects. The imagological tumor (T) staging, the extramural depth (EMD) of the cancer tissues, and the extramural vascular invasion (EMVI) grading were analyzed. A dense residual single-axis super-resolution network model was also constructed for enhancing CT images. It was found that the CT images processed using the algorithm were clear, and the peak signal-to-noise ratio (PSNR) and structural similarity (SSIM) were 33.828 dB and 0.856, respectively. In the imagological T staging of CC patients, there were 17 cases in the T3 stage and 68 cases in the T4 stage. With the EMD increasing, the preoperative carcinoembryonic antigen (CEA) highly increased, and the difference was statistically significant (*P* < 0.05). The postoperative hospital stays of patients were also different with different grades of EMVI. The hospital stay of grade 1 patients (19.45 days) was much longer than that of grade 2 patients (13.19 days), grade 3 patients (15.36 days), and grade 4 patients (14.36 days); the differences were of statistical significance (*P* < 0.05). It was suggested that CT images under the deep learning algorithm had a high clinical value in the evaluation of T staging, EMD, and EMVI for the diagnosis of CC.

## 1. Introduction

Colorectal cancer (CC) is one of the most common malignant tumors clinically, ranking third in the incidence of malignant tumors and fourth in the mortality of malignant tumors around the world [1, 2]. About 1.2 million people suffer from CC every year, and about 600,000 people lose their lives as a result. Its incidence is higher in Europe and America, but lower in Asia; the incidence is higher in men than in women [3]. In China, the incidence of CC in urban areas and rural areas is about 31.29/105 and 16.99/105, respectively, ranking third and sixth among malignant tumors. In urban areas, the 5-year survival period of CC patients is about 50%; in rural areas, the 5-year survival period is less than 40% [4, 5]. So far, even if there is no specific statistical data on the incidence of CC, the incidence of CC in China has shown an increasing trend year by year due to the changes in people's living standards in recent years and the westernization of living habits. Surgery is still the most effective way to treat CC [6]. Nonperitonealized CC refers to a tumor located on the posterior wall of the ascending colon or descending colon, where the posterior wall of the intestine is not covered by the peritoneum [7]. With the clinical staging and diagnosis of nonperitonealized CC, there have been many studies on the circumferential resection margin (CRM) of this disease. However, the CRM is caused by a sharp separation during surgical resection. The scope of surgical resection has a great influence on it, and it cannot truly reflect the infiltration of cancer tissues [8]. For the development and popularization of complete mesorectal excision (CME) surgery in China, the scope of surgical resection is more thorough than that of traditional radical surgery, significantly improving the surgery quality. On the contrary, the CRM of some deeply infiltrated cancer tissues may still be negatively affecting the correct evaluation of the CRM [9].

As one of the most commonly used clinical imaging techniques, computerized tomography (CT) has been applied in the examination of various diseases. Plain CT scanning is the basis of all CT examinations, with a relatively simple operation and a faster scanning speed. Most diseases can be detected without injection, such as craniocerebral hemorrhage, cerebral infarction, brain tumor, and fracture. Enhanced CT scanning is a further examination of plain CT scanning. When an issue is found in plain CT scanning, enhanced CT scanning is required; it is clearer than plain scanning to evaluate the blood supply of the lesions [10]. In recent years, deep learning has made great achievements in digital image processing. The deep convolutional neural network can learn the structural feature information of high and low frequencies in complex images, and it can give better results than traditional algorithms in the processing of medical images. The three-dimensional reconstruction algorithm of tomographic images eliminates the volume effect to a certain extent on the basis of deep learning, while maintaining the isotropy of the volume data [11, 12]. In the method of edge super-resolution depth slice interpolation, some scholars proposed a single-axis super-resolution method by combining the content of the orthogonal direction of the slice to obtain the reconstructed imaging image of the coronal plane and the sagittal plane [13]. From this, the idea of developing a three-dimensional reconstruction of abdominal CT images under the single-axis super-resolution reconstruction algorithm came up. It aimed to reduce the radiation intensity of CT tomographic images and improve the quality of the three-dimensional reconstruction model.

With CT imaging principles and deep learning-based super-resolution reconstruction technology, it was proposed to construct a feature-enhanced residual dense network model with a single axis and super-resolution [14]. The uniaxial super-resolution was utilized to perform three-dimensional reconstruction of abdominal CT images and evaluate its application value in the diagnosis of nonperitonealized CC trying to provide a certain theoretical basis for the clinical diagnosis of the disease.

## 2. Data and Methods

### 2.1. General Data of Patients

One hundred and three patients with nonperitonealized CC admitted to the hospital from March 2018 to March 2020 were included. Their general information and clinicopathological information were collected and recorded. The patients consisted of 49 males and 54 females, with an average age of 67.67 ± 14.32 years. There were 85 cases with lesions in the ascending colon and 18 cases with that in the descending colon. This study had been approved by the ethics committee of the hospital. The patients and their family members fully understood the status and signed the informed consent forms.

Inclusion criteria were as follows: patients who were diagnosed with CC according to the pathological diagnosis; patients who were in phase II of the pathological staging; patients who underwent the colon surgery for the first time; the surgery for patients was radical resection.

Exclusion criteria were as follows: patients who underwent emergency surgery; patients who died during the perioperative period; patients who had cachexia complications, or did not have the complete data.

### 2.2. CT Examination and Image Processing

The 64-slice spiral CT instrument was used for examinations. Patients were asked to fast for 4 hours before the examination, and drink 1L of purified water 30 minutes before the examination. The injected contrast agent was an iohexol injection with a dose of 80–100 mL, and the injection speed was 3 mL per second. CT scanning was performed 30 seconds after the start of the injection. The scanning range was from the top of the diaphragm to the plane of the pubic symphysis; the slice thickness was 5 mm, the scanning voltage was 125–145 kV, the scanning current was 150–220 mA, the interval was 0.984 : 1, and the scanning time was about 5 minutes. During the process, patients needed to minimize their movements, reduce mood swings, and calm their breathing. Finally, the obtained image data were uploaded to the computer, and the dense residual single-axis super-resolution network algorithm was applied to process the images.

### 2.3. Evaluation of the Results

For the imagological tumor (T) staging of patients, 2 radiologists with rich clinical imaging experience were invited, and the multislice spiral CT instrument was used [15]. The staging standards are shown in Table 1.

In the process of reading the images, both radiologists divided the stages with the independent double-blind principle. If the staging results of the two radiologists were different, a third radiologist was asked to stage. If the result of the third radiologist was the same as that of one of the first two radiologists, the stage was determined.

The imagological grading standards of extramural vascular invasion (EMVI) [16] are shown in Table 2 for details.

Both radiologists determined the EMVI grading independently and double-blindly. If the two results were different, a third radiologist was involved. When the result of the third radiologist was the same as that given by one of the first two radiologists, the EMVI grade was confirmed.

Two radiologists were invited to measure the extramural depth (EMD) of cancer tissues. The vertical distance between the cancer tissue outside the farthest end of the intestinal wall and the intestinal wall was measured, and the EMD was calculated by taking the average value.

### 2.4. Model Construction of Dense Residual Single-Axis Super-Resolution Network

The original low-resolution image *E*^LR^ is the input, and a multiscale feature convolution layer was used to extract the shallow mapping layer, which is expressed as

(1) $$\begin{array}{c} M_{-1}=G_{SFE1}\left(E^{LR}\right). \end{array}$$

*G*_*SFE*1_ represents a convolution operation of the multiscale feature extraction. Then, it came to the deep feature learning part of the dense residual block for further extraction of the shallow map and the global residual map. It is expressed as

(2) $$\begin{array}{c} M_{0}=G_{SFE2}\left(M_{-1}\right). \end{array}$$

*G*_*SFE*2_ is the convolution operation of the secondary shallow feature extraction, and *M*_0_ is taken as the input of the deep feature extraction module. Thus, (3) is obtained.

(3) $$\begin{array}{c} M_{\text{n}}=G_{R\;DB\;,\text{n}}\left(M_{\text{n}-1}\right)=G_{R\;DB\;,\text{n}}\left(G_{R\;DB-1}\left(\ldots \left(G_{R\;DB\;,1}\left(M_{0}\right)\right)\ldots \right)\right). \end{array}$$

*G*_*R*  *DB*  ,*n*_ is the nth dense residual block. *G*_*R*  *DB*  ,*n*_ is the iterative nesting method, including convolution and activation functions. Each dense residual block had a corresponding *M*_*n*_; therefore, *M*_*n*_ refers to the local information in the feature map.

Afterward, the staircase features of a series of dense residual blocks were learned, and the results of all layer features extracted in the feature extraction stage were used for global residual learning and global dense feature fusion. The features of different layers were merged using Concat and convolution operations, and then, they were merged for dense feature fusion (DFF). In this case, DFF could be expressed as

(4) $$\begin{array}{c} M_{\text{DF}}=G_{\text{DFF}}\left(M_{-1},M_{0},M_{1},\ldots ,M_{D}\right). \end{array}$$

*F* refers to the feature image after depth mapping. It was sent to the feature enhancement module to obtain the final feature image, which is expressed as

(5) $$\begin{array}{c} M_{LR}=G_{FE}\left(M_{LR-1}\right). \end{array}$$

*G* stands for the operation of weighted reinforcement learning in the image. When the feature extraction ended, the feature map *M* was regarded as the multiscale upsampling layer *VM-Meta* of any scaling factor. It is described as

(6) $$\begin{array}{c} M_{SR}=G_{V-MMeta}\left(M_{LR}\right). \end{array}$$

In (6), *G* is the upsampling operation. The size of the feature image in the vertical axis direction became *n* times of that of the input image (*n* represented the magnification times). 3 × 3 convolution was used to perform the feature compression operation, which is shown as

(7) $$\begin{array}{c} E_{SR}=G_{REC}\left(M_{SR}\right). \end{array}$$

The output of the model was thus obtained, that was, the high-resolution image *SR*.

In the model optimizer, the *L1* loss function was adopted for {*E*_*LR*_^*i*^, *E*_*HR*_^*i*^}_*i*=1_^*K*^ of *K* pairs of data that were taken as the training set. The loss function was then expressed as

(8) $$\begin{array}{c} L\left(\gamma \right)=\frac{1}{K}\sum_{i=0}^{K}|G_{\text{model}}\left(E_{LR}^{i}\right)-E_{HR}^{i}|. \end{array}$$

*γ* is the network parameter set optimized using the Adam optimizer algorithm.

### 2.5. Statistical Analysis

SPSS22.0 was used to perform statistical analysis on the data. Quantitative data were compared using the *χ*^2^ test or the Fisher exact probability method. The relationship among variables was shown by correlation analysis, and the consistency evaluation was performed by the Kappa test. When *P* < 0.05, it meant that the difference was of statistical significance.

## 3. Results

### 3.1. General Information

**Figure 1:**
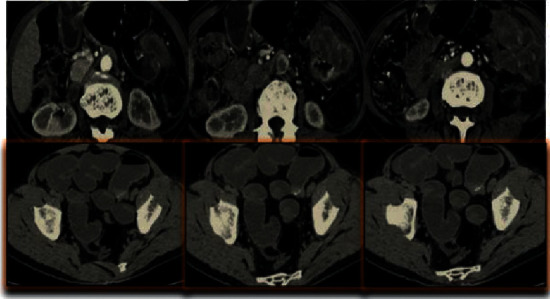
CT images of the patients before being processed by the single-axis super-resolution algorithm. All were enhanced arterial-phase scanning images.

**Figure 2:**
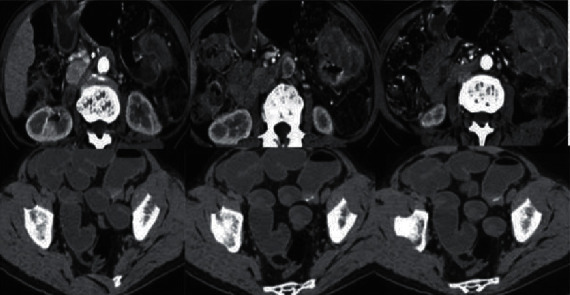
CT images of the patients after being processed by the single-axis super-resolution algorithm.

**Figure 3:**
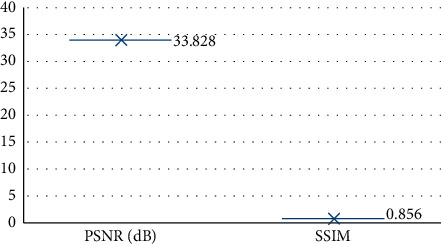
PSNR and SSIM of the obtained images after being processed by the single-axis super-resolution algorithm.

Figures 1–3 show some CT images of the patients before and after being processed by the single-axis super-resolution algorithm, as well as the structural similarity (SSIM) and peak signal-to-noise ratio (PSNR) of the processed images.

From Figures 1–3, the CT images of patients were manifested with obvious thickening of the colorectal wall, showing irregular localization or diffuse thickening. Obvious enhancement was observed using CT-enhanced scanning, with colonic lumen stenosis and low-density necrotic zones inside the cancer tissues. After super-resolution reconstruction, the obtained images were clearer, and the PSNR and SSIM were 33.828 dB and 0.856, respectively.

### 3.2. Imagological T Staging

**Figure 4:**
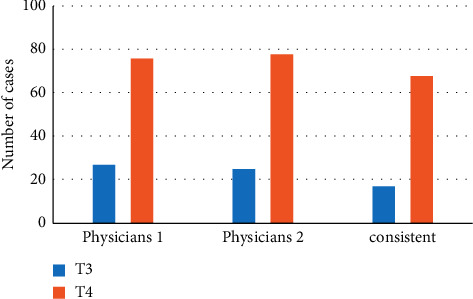
T staging results of imaging.

Figure 4 shows the imagological T staging results of the 103 patients with CC, which were determined by two radiologists. It was found that all the staging results of the two radiologists were in the T3 and T4 stages, having nothing to do with T1 and T2 stages. Radiologist 1 gave the results of 27 cases in the T3 stage and 76 cases in the T4 stage. Radiologist 2 gave the results of 25 cases in the T3 stage and 78 cases in the T4 stage. The consistency analysis of the results of the two radiologists was made for the common staging results, which shows 17 cases in the T3 stage and 68 cases in the T4 stage. From the Kappa consistency analysis, it was found that the imagological T staging of the two radiologists had generally poor consistency (*κ* = 0.499, *P* < 0.05).

**Figure 5:**
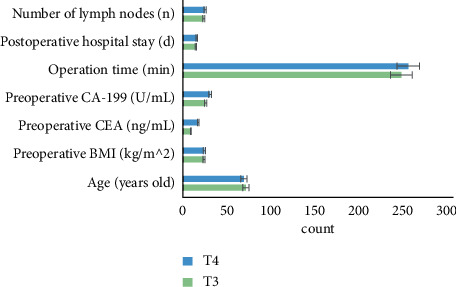
Comparison of clinical data of imagological T staging.

As shown in Figure 5, the patients were divided into the T3 stage group and the T4 stage group according to imagological T staging. Their age, preoperative body mass index (BMI), preoperative carcinoembryonic antigen (CEA), preoperative carbohydrate antigen-199 (CA-199), time of surgery, postoperative hospital stays, and the statistical analysis results of lymph node examinations were compared. It was shown that all the terms of the T3 stage group were less than those of the T4 stage group, but the differences between the two groups were not statistically significant (*P* > 0.05).

### 3.3. EMD of Cancer Tissues

**Figure 6:**
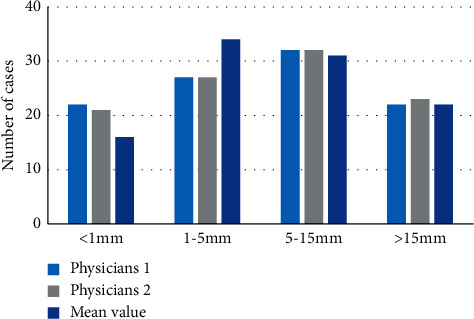
Evaluation results by the two radiologists on the EMD.

Figure 6 shows the imagological EMD measurement of 103 patients with CC by 2 radiologists. For the EMD measurement results of radiologist 1, EMD <1 mm was observed in 22 cases, 1 < EMD < 5 mm in 27 cases, 5 < EMD < 15 mm in 32 cases, and EMD >15 mm in 22 cases. For those of radiologist 2, there were 21, 27, 32, and 23 cases with EMD <1 mm, 1 < EMD < 5 mm, 5 < EMD < 15 mm, and EMD >15 mm, respectively. The Kappa method was adopted to analyze the consistency of the results of the two radiologists, and it was found to be generally poor (*κ* = 0.384, *P* < 0.05). The average values of the results of both doctors were taken, thus, there were 16 cases with EMD <1 mm, 34 cases with 1 < EMD < 5 mm, 31 cases with 5 < EMD < 15 mm, and 22 cases with EMD >15 mm.

**Figure 7:**
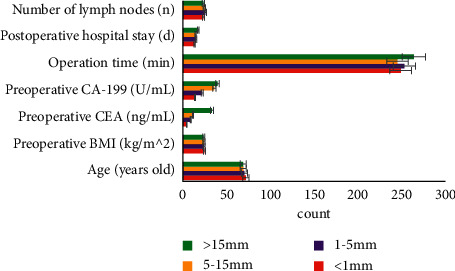
Comparison of clinical pathological data in imagological EMD.

According to the imagological measurement results of the EMD, the patients were divided into 4 groups, which were <1 mm group, 1–5 mm group, 5–15 mm group, and >15 mm group. The age, preoperative BMI, preoperative CEA, preoperative CA-199, time of surgery, postoperative hospital stays, and the number of lymph nodes were obtained. As shown in Figure 7, with the increase of the EMD, the preoperative CEA also increased, and the differences were statistically significant (*P* < 0.05). Through the correlation test, it was found that there was a certain correlation between the EMD and preoperative CEA (*P* < 0.05).

### 3.4. EMVI Evaluation Results

**Figure 8:**
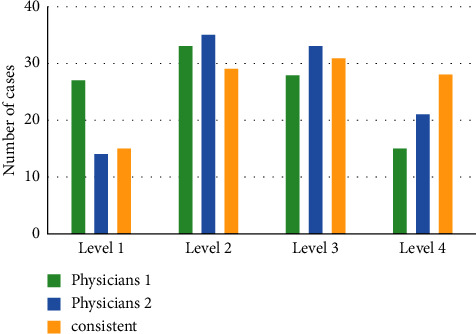
Imagological EMVI evaluation results by two radiologists.

Figure 8 shows the imagological EMVI evaluation results of 103 patients with CC made by the two radiologists. The EMVI results of radiologist 1 are as follows: 27 cases were judged in grade 1, 33 cases in grade 2, 28 cases in grade 3, and 15 cases in grade 4. As for the EMVI results of radiologist 2, 14, 35, 33, and 21 cases were in grade 1, grade 2, grade 3, and grade 4, respectively. The consistency of the EMVI results of radiologist 1 and radiologist 2 was analyzed using the Kappa method, and it was found to be poor (*κ* = 0.281, *P* < 0.05). The final EMVI results are as follows: 15, 29, 31, and 28 cases were in grade 1, grade 2, grade 3, and grade 4, respectively.

**Figure 9:**
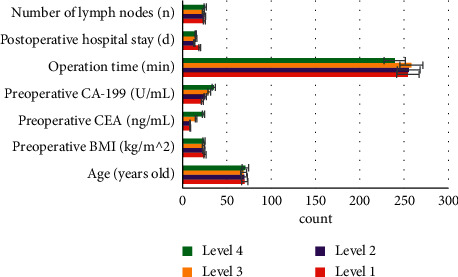
Comparison of clinical pathological data in imagological EMVI.

With the evaluation results of EMVI, Figure 9 shows the comparisons of the age, preoperative BMI, preoperative CEA, preoperative CA-199, time of surgery, postoperative hospital stays, and the number of lymph nodes. There were differences in the postoperative hospital stays of patients in different EMVI grades, which were statistically significant (*P* < 0.05). However, no correlation was found between the EMVI grading and the postoperative hospital stays (*P* < 0.05) via the correlation test.

## 4. Discussion

The clinical examinations of CC patients generally include a barium meal, fiber colonoscopy, and imaging examinations [17]. A literature suggested that intestinal barium meal examination can show the characteristics of intestinal mucosal injury clearly, but there are certain limitations in showing changes in the outer lumen and the intestinal wall [18]. With fiber colonoscopy, the color of the intestinal mucosa and ulcers or bleeding on the surface of the intestinal mucosa can be observed directly. For suspected lesions, the tissues can be directly taken for medical examination. But the lesions outside the intestinal cavity and the infiltration of the surrounding tissues and organs cannot be shown [19].

Some scholars have proposed that CT scanning can obtain the data of the entire abdomen of patients and process them. It can not only clarify the location of the number of lesions, the situation inside and outside the intestinal cavity of the lesion, the invasion of adjacent organs and tissues, and the distant metastasis, etc.; it can also perform the staging of tumors [20, 21]. To improve the quality of CT images, the dense residual single-axis super-resolution network model was also constructed for the processing of CT images. The processing performance of this algorithm was analyzed, and the CT images obtained by this algorithm were clearer, with the PSNR of 33.828 dB and SSIM of 0.856. These indicated that the algorithm designed in this research had a better performance in processing CT images, worthy of promotion and application. It was shown from data analysis that, for CT imagological T staging of CC patients before surgery, there were 17 cases in the T3 stage and 68 cases in the T4 stage. The age, preoperative BMI, preoperative CEA, preoperative CA-199, time of surgery, postoperative hospital stays, and the number of lymph nodes of patients were analyzed. From the statistical analysis results, all the parameters in the T3 stage were less than those in the T4 stage. For the EMD of cancer tissues, EMD <1 mm was observed in 16 cases, 1 < EMD < 5 mm in 34 cases, 5 < EMD < 15 mm in 31 cases, and EMD >15 mm in 22 cases. Meanwhile, the preoperative CEA also increased with the increasing EMD, and the difference was statistically significant (*P* < 0.05). By the correlation test, it was discovered that there was a certain correlation between the EMD and preoperative CEA (*P* < 0.05). It was indicated that CT examination had a high clinical value for the imagological staging of CC and the diagnosis of cancer tissue invasion, which was exactly similar to the results of Flor et al. [22].

In the diagnosis of EMVI, 15 cases were assessed in grade 1, 29 cases were in grade 2, 31 cases were in grade 3, and 28 cases were in grade 4. The patients in different grades of EMVI had different hospital stays after surgery, and the differences were statistically significant (*P* < 0.05). However, the correlation test found that the EMVI grading had no correlation with the postoperative hospital stays (*P* < 0.05). Therefore, CT examination had a great clinical value for the diagnosis of CC invasion before surgery. There are also studies that show that the diagnostic efficiency and the sensitivity of EMVI evaluation with imaging are relatively low. For example, poor accuracy is obtained in the preoperative evaluation of tumors, so that it is of no clinical practical significance [23]. Some researchers believe that, even though imaging is less sensitive to the diagnosis of EMVI, it has a positive significance in the diagnosis of adjacent vascular infiltration of cancer tissues, showing an important significance in improving the prognosis of the disease [24].

## 5. Conclusion

The uniaxial super-resolution algorithm was used to perform three-dimensional reconstruction of abdominal CT, and its application value in the diagnosis of nonperitonealized CC was evaluated by analyzing the imagological T staging, the EMD of cancer tissues, and the EMVI grading. It was concluded that the CT images processed by the deep learning algorithm were clearer, and the PSNR and SSIM were measured as 33.828 dB and 0.856, respectively. CT can do well in preoperative staging of nonperitonealized CC, imagological T staging, and the evaluation of EMD and EMVI, having a high clinical value in the diagnosis of CC. However, some shortcomings were also exposed. The sample size was relatively small, and there was not a prospective experiment with large samples. In the future, the sample should be expanded for further research. Altogether, the results provided a theoretical basis for the clinical imaging diagnosis of nonperitonealized CC.

## Figures and Tables

**Table 1:** T staging of nonperitonealized CC imaging.

T staging	Specific manifestations
T1 stage	There was no significant imaging change in the intestinal wall of the diseased side
T2 stage	The intestinal wall of the diseased side was thickened asymmetrically, protruding into the intestinal cavity. But the muscular layer of the intestinal wall was smooth and contiguous, and the surrounding adipose tissues did not change.
T3 stage	The intestinal wall was smooth or thickened by the nodular discontinuously, the muscle layer of the lesion was discrete, and the adjacent adipose tissues were infiltrated
T4 stage	Cancer tissues infiltrated the muscular layer to the peritoneum, or the anterior edge of cancer nodules infiltrated adjacent organs (when the distance between the cancer tissue and the fused fascia was less than 1 mm, it was also present in the T4 stage).

**Table 2:** Imagological grading of EMVI for CC patients.

EMVI grading	Specific manifestations
Grade 1	No obvious EMVI occurred
Grade 2	The blood vessels adjacent to the cancer tissues became slightly curved
Grade 3	The small blood vessels adjacent to the cancer tissues showed nodular changes (the nodular manifestations had the same enhancement degree within the small blood vessels and the cancer tissues)
Grade 4	The large blood vessels adjacent to the cancer tissues were significantly infiltrated (tumor embolus could be observed in large enhanced veins)

## Data Availability

The data used to support the findings of this study are available from the corresponding author upon request.
